# Pre-Fermentation Water Addition to High-Sugar Shiraz Must: Effects on Wine Composition and Sensory Properties

**DOI:** 10.3390/foods9091193

**Published:** 2020-08-28

**Authors:** Bo Teng, Paul R. Petrie, Damian Espinase Nandorfy, Paul Smith, Keren Bindon

**Affiliations:** 1 College of Science, Shantou University, Shantou 515063, China; bteng@stu.edu.cn; 2 The Australian Wine Research Institute, P.O. Box 197, Glen Osmond, SA 5064, Australia; paul.petrie@sa.gov.au (P.R.P.); damian.espinasenandorfy@awri.com.au (D.E.N.); paul.smith@wineaustralia.com (P.S.); 3 South Australian Research and Development Institute, Waite Research Precinct, Urrbrae, SA 5064, Australia; 4 School of Agriculture, Food and Wine, Waite Research Institute, University of Adelaide, Glen Osmond, SA 5064, Australia; 5 School of Mechanical and Manufacturing Engineering, University of New South Wales, Sydney, NSW 2052, Australia; 6 Wine Australia, Industry House-National Wine Centre, Cnr Hackney and Botanic Roads, SA 5000, Australia

**Keywords:** phenolics, alcohol, colour, tannin, polysaccharide, fermentation, volatile, dark fruit, red fruit, dried fruit, hotness, brown, Quantitative descriptive sensory analysis (QSDA)

## Abstract

Changes to Australian regulations now allow the limited addition of water to high-sugar musts pre-fermentation. In light of these changes, this study explored how water addition affects Shiraz wine composition and sensory properties. Wines were made from grapes at ≈13.5, 14.5 and 15.5° Baume. Water was added to musts from the ripest fruit by direct addition, or by using a juice substitution (run-off and replace) approach. To compare the effect of juice run-off independently, saigneé treatments were included. Wines made from the fruit that was harvested earlier generally had a lower “opacity” and higher “red fruit” aroma as the defining sensory attributes. Undiluted wines made from riper fruit had higher phenolics, and were characterised by “dark fruit” and “dried fruit” attributes, and “spice”, a “brown colour” and “opacity”. These attributes were accentuated in wines from the same fruit which received saigneé treatments and reduced in all of the water addition treatments. In particular, higher levels of water addition without juice substitution increased the “cooked vegetable” and “drain” attributes in the wines. This indicates possible negative effects of larger water additions, such that a low to moderate adjustment in Shiraz winemaking is suggested.

## 1. Introduction

Wine production in Australia, as in many other parts of the world, is increasingly under pressure due to the effects of vintage compression. Vintage compression can be defined as a narrowing of the period within which grapes ripen, and is thought to be primarily due to warming climatic conditions [1]. As a result, the logistics of production can come under pressure, since grape varieties which historically ripened in a staggered manner, and over a wider period, reach commercial ripeness within a similar time frame. In practice, limits on the availability of harvesters, or winery tank space, can result in decisions to delay the harvesting of fruit. A natural consequence of this delay is an increase in the sugar concentration of harvested grapes [2], potentially exacerbated by a loss in berry volume, i.e., fruit dehydration or shrivel [3,4]. Higher sugar musts lead not only to undesirably high alcohol levels in wine, but can result in a stress response in yeast leading either to the cessation of fermentation (“stuck” fermentation) or the production of elevated levels of yeast metabolites such as acetic acid, which potentially decrease wine quality [5].

In response to the above-mentioned concerns, changes in Australian legislation in 2017 authorised the addition of limited quantities of water to must (pre-fermentation, and to reduce the must sugar concentration to no lower than 13.5° Baume, or 24.4° Brix), which has led to an increased interest within the research community in recent years to understand the effects of water addition on wine composition and quality [6,7,8]. Initial studies on red wine production undertook water addition by applying a juice substitution approach, in which juice was removed (“run off”), and replaced with an equal volume of water in order to maintain the ratio of grape solids to liquid [3,9]. In a Cabernet Sauvignon must, it was found that water addition had minimal effects on wine phenolics, volatiles and sensory properties, even at very high levels of substitution (32%). A follow-up study aiming to compare juice substitution outcomes in Cabernet Sauvignon and Shiraz found a divergence in response between the two grape varieties [7]. In that study, higher levels of water substitution (25%) increased the colour and tannin in Cabernet Sauvignon wines and decreased these measures in Shiraz, while minor effects were found for lower quantities of water addition (6–10%). Further work by our group confirmed that for the Shiraz variety, water addition either by substitution or direct addition decreased wine tannin and colour, even at low addition rates of ≈8%, and that colour measures in particular decreased further as water addition increased (14–16%) [6]. Interestingly, our results showed that the way in which water was applied, by direct addition or substitution, did not affect outcomes on wine colour or tannin. This indicated that for a limited range of water addition, the juice:solids ratio did not affect the extraction or retention of phenolics. A further study [8] also compared substitution and direct addition techniques for Shiraz must, but on less mature fruit (12.6 and 14° Baume) than was used in our previous report (15.6° Baume) [6]. That work showed that at lower rates of water addition (12%), the wine tannin concentration was not significantly affected, but the wine tannin concentration decreased as water addition was increased up to 47%. Contrary to what was found in our earlier study [6], direct water addition was shown to amplify the reduction in tannin concentration at higher water addition rates when compared to the substitution approach. For wine colour, it was found that the effect of water addition was dependent upon fruit maturity. Greater and more consistent losses in colour with water addition were found in wines made from riper grapes, and the result was greater when water was added directly rather than substituted [8]. This work provided support for our previous observations in even riper fruit [5], and indicated that there may be a ripening-dependent effect in Shiraz grapes, by which losses in colour (and potentially tannin concentration) in response to water addition are exacerbated in wines as grape maturity advances.

A number of unknowns still exist for the water addition process in Shiraz winemaking. Previous reports which provided information on the sensory outcomes of water addition in Shiraz wines focused on grapes which were within an expected range of commercial ripeness [7,8] at ≤14.5° Baume. As yet, data are not yet available on the sensory outcomes of water addition to Shiraz must from fruit harvested at higher must sugar concentrations, which might be considered to be a problematic consequence of vintage compression, hence definitively requiring the intervention of a pre-fermentative water addition. Previous work using water substitution in a 14.5° Baume Shiraz must showed a consistent loss of higher alcohol acetate esters, and certain ethyl esters, in particular hexyl acetate, 3-methylbutyl acetate and phenylethyl acetate [7], which were somewhat independent of the quantity of water added. Despite this, it was found that the change in sensory properties with must substitution was minor in Shiraz [7], and only wine astringency and hotness (alcohol) were significantly reduced at higher addition rates (25%). On the other hand, the comparison of must substitution and direct addition to add water to a 14° Baume Shiraz must [8] showed only minor or no changes to wine sensory properties when the addition was low (10%). However, when the addition was high (47%), the intensity of key sensory attributes such as astringency, hotness, dark fruit aroma and flavour all decreased. For all of the above-mentioned studies, the smaller water additions were more appropriate to the specification defined by the recent changes in legislation and as may be practiced in commercial winemaking worldwide, therefore it is unlikely that a substitution of juice with water or a direct addition of water would ever proceed to the higher levels defined in some published works [3,7,8,9].

In light of these findings, it was considered relevant to revisit the effect of water addition and substitution in very ripe Shiraz fruit (total soluble solids, TSS, approximately 28° Brix, 15.5° Baume), particularly considering that marked changes in the wine volatile and flavour profile are expected as the fruit enters the shrivel phase [10]. Since the increase in TSS, and expected wine alcohol associated with extended “hang time” can be legally overcome by water addition in Australia and in other wine producing countries, it becomes critical to establish whether this practice can be justified in terms of wine sensory outcomes. This becomes particularly relevant in light of a recent observation made for Cabernet Sauvignon wines prepared from over-ripe, shrivelled fruit [9]—a “port-like” attribute developed which was neither compensated for, nor removed, by water substitution.

To address these questions, wines were prepared from high TSS Shiraz fruit with different levels of water substitution or addition applied, and bottled wines were subjected to a compositional and sensory analysis 12 months after the completion of fermentation. By way of comparison, wines were also prepared from grapes harvested at two earlier time points, at 13.5° Baume and 14.5° Baume in order to draw conclusions on the value of extending “hang time” (and therefore requiring pre-ferment water addition) as opposed to harvesting at a target ripeness level. A previous report on phenolic outcomes has been published for young wines [6], and the results presented in this paper aimed to extend the preliminary findings.

## 2. Materials and Methods

### 2.1. Grapes and Wines for the Ripening and Water Addition Trial

Shiraz grapes were harvested from a research vineyard in Nuriootpa, Barossa Valley, South Australia, in the 2017 season. Hand-harvesting was performed at three successive dates at a target TSS of 13.5, 14.5 and 15.5° Baume, and designated H-1, H-2 and H-3, respectively. The procedure for harvesting, fruit randomisation and winemaking has been reported in full previously [6]. Briefly, the winemaking treatments conducted in triplicate for the H-3 fruit were as follows, with pure rainwater additions as follows, calculated based on an expected liquid extraction rate of 65% (30 L per 45 kg of grapes):

- DA-1: Direct addition of 5 L of water to the must
- DA-2: Direct addition of 2.4 L of water to the must
- RR-1: Juice substituted with 5 L of water
- RR-2: Juice substituted with 2.4 L of water
- RO-1: Removal of 5 L of juice without water addition
- RO-2: Removal of 2.4 L of juice without water addition

Based on the concentration of yeast assimilable nitrogen (YAN) in each treatment following dilution, the YAN concentration was adjusted to a total of 300 mg/L with diammonium phosphate. Each must was also adjusted to a pH of 3.8 with tartaric acid to match that of the earliest harvest, prior to inoculation with yeast. Each treatment was inoculated with *S. cerevisiae* yeast (EC1118, Lallemand, Montréal, QC, Canada, 500 ppm) on the first day after crushing, and followed sequentially by an addition of lactic acid bacteria, *O. oeni* (VP41, Lalvin, St. Simon, Paris, France) on the second day after crushing. During fermentation, the cap of each ferment was plunged two times each day, and alcoholic fermentation was monitored by the consumption of sugar. Fermentation on skins was carried out over 9 days at 20 °C and then each treatment was pressed. In pressed treatments, fermentation continued at 20 °C until dryness was reached (residual sugar <1 g/L) and were held at 20 °C until malolactic fermentation was complete (malic acid <0.1 g/L). Wine glucose, fructose and malic acid were determined using enzymatic methods and an automated analyser (Daytona, Randox Laboratories, Crumlin, United Kingdom) to confirm the completion of alcoholic and malolactic fermentation. Wines received 80 ppm of SO_2_ and were cold-settled at 0 °C for a minimum of 28 days, then adjusted to a final titratable acidity of 6 g/L with tartaric acid, and a free SO_2_ of 45 ppm. Wines were filtered by cross-flow filtration, and bottling in 750 mL glass bottles under screw-cap occurred in November 2017.

### 2.2. Wine Compositional Analysis

The compositional analysis of the experimental wines was scheduled to coincide with the sensory analysis, which took place when the wines had been ≈5 months in-bottle (≈12 months post-fermentation). For the analysis of phenolic compounds, wine and must samples were centrifuged for 5 min at 16.1 *g* prior to analysis. The tannin concentration and colour composition were analyzed in wines using a standard high-throughput method [11], at a minimum of duplicate analyses according to the precise protocol outlined previously [6]. Tannin was isolated by a solid phase extraction [12] and a gel permeation chromatography analysis of the extracts was conducted following the original published protocol [13] with the modifications reported previously [14]. The purified tannin fractions were also analysed by phloroglucinolysis [15] using a published high-performance liquid chromatography (HPLC) approach [13]. Wine samples were also analysed for polymeric pigments according to a published HPLC method [11] and polymeric pigments were quantified as malvidin-3-O-glucoside units using a commercial standard (Polyphenols Laboratories, Sandnes, Norway).

Yeast volatile fermentation products were analysed using stable isotope dilution analysis in conjunction with headspace solid-phase microextraction coupled with gas-chromatography mass spectrometry (GCMS) as described previously [16] and twenty-seven compounds, including ethyl and acetate esters, higher alcohols and volatile acids, were quantified.

For the analysis of polysaccharides, a 1 mL aliquot of wine was added to 5 mL of absolute ethanol and precipitated at 4 °C for 18 h. Samples were centrifuged at 8000 *g* for 5 min, the supernatant was discarded and the pellet retained. Pellets were washed with 5 mL ice-cold 80% *v*/*v* ethanol, recentrifuged, and the recovered pellet briefly air-dried to remove excess ethanol. Pellets were reconstituted in water, frozen, lyophilized and then reconstituted in 2 M trifluoroacetic acid prior to hydrolysis at 100 °C for 3 h. Hydrolysates were cooled on ice, concentrated under vacuum at 30 °C, and resuspended in water. The monosaccharides released from polysaccharides following hydrolysis were quantified using an adaptation of a published method [17] with the modification for the derivatisation, recovery and analysis of monosaccharide adducts performed as described previously [18]. The polysaccharides were quantified as the sum of the monosaccharides.

Residual amino acids in wines were also derivatised according to a previously-described protocol [19] and quantified by HPLC following a 1:10 dilution with water with the following modifications: derivatised wine samples were injected into an Agilent 1260 UHPLC equipped with a Trajan C18 120Å 5 µm (250 mm × 4.6 mm) column (Trajan, Ringwood, VIC, Australia). Separation was achieved with a solvent system of 50 mM ammonium acetate pH 6.5 (Solvent A) and 100 mM ammonium acetate/acetonitrile pH 6.5 (Solvent B) at a flow rate of 2 mL/min with a linear gradient reaching 70% of Solvent B at 45 min, 100% of Solvent B at 48 min and then re-equilibrated for 60 min to starting conditions. The standards for identification and quantification were purchased from Sigma-Aldrich Pty Ltd. (a subsidiary of Merck, Macquarie Park, NSW, Australia).

### 2.3. Sensory Analysis

Initial bench tasting by a panel of expert technical wine assessors resulted in no exclusion of any fermentation replicates due to off-flavours or winemaking artefacts. Each of the winemaking replicate wines were evaluated using quantitative sensory descriptive analysis (QSDA). An additional nine wines from a closely related study, made from grapes from the same vineyard, were also evaluated as part of the sensory assessment (data not shown). A panel of eleven assessors (nine females, two males) with an average age of 49 years (SD = 9.5) was convened to evaluate the wines. The assessors attended three two-hour training sessions to determine suitable descriptors for rating in the formal sessions. All the wines from the study were progressively presented during training sessions to generate and refine appropriate descriptive attributes and definitions through a consensus-based approach. Wines were evaluated by appearance, aroma and palate. In the second session, reference standards for aroma, basic taste and mouthfeel attributes were presented and discussed. The sensory reference standards were evaluated in all subsequent sessions. Following the third training session, assessors participated in a practice session in the sensory booths under the same conditions as those for the formal sessions. After the practice session, any attributes and definitions which needed adjustment were discussed and the final list of terms and the standards were finalized. Samples were presented to panelists in 30 mL aliquots in 3-digit-coded, covered, ISO standard wine glasses at 22–24 °C, in isolated booths under daylight-type fluorescent lighting. A randomized presentation order was followed except in the practice sessions when there was a fixed presentation order. All samples were expectorated. The assessors were presented with four trays of three wines per tray. The assessors were forced to have a 60-s rest between samples and were encouraged to rinse with water, and a minimum ten-minute rest between trays. During the ten-minute break assessors left the booths. The formal evaluation was completed in three two-hour sessions on separate days. The wines were presented to assessors two times, in a modified Williams Latin Square incomplete random block design generated by Fizz sensory acquisition software (version 2.51, Biosystems, Cousteron, France). A new bottle was used for each of the assessment days. The intensity of each attribute was rated using an unstructured 15 cm line scale (numericized 0 to 10), with indented anchor points of “low” and “high” placed at 10% and 90%, respectively. The data were acquired using Fizz sensory software (Version 2.47B, Biosystems, Cousteron, France).

### 2.4. Statistical Analysis of the Wine Composition and Sensory Results

Analysis of variance (ANOVA) and post-hoc means comparison tests were carried out using Minitab 18 (Minitab Inc., Sydney, NSW, Australia) for sensory data and JMP 14 (JMP Australia and New Zealand, Lane Cove, NSW, Australia) for wine chemical data. For the sensory data, the effect of the treatment, judge, judge by treatment, winemaking replicate nested into treatment, judge by winemaking replicate nested into treatment, and presentation replicate nested into treatment and winemaking replicate were assessed for each attribute, treating judge as a random effect (Table S1). A principal component analysis (PCA) was performed using XL STAT (XLSTAT 2020.3, Paris, France) and partial least squares (PLS) regression analysis was performed using the Unscrambler 11 (CAMO Software, Oslo, Norway) software package. All PCA and PLS analyses were performed with a full cross validation.

## 3. Results and Discussion

### 3.1. Outcomes of the Harvest Date, Water Addition and Saigneé Treatments on Wine Composition

Up to the point of bottling, the titratable acidities of the final wines were successfully standardised at 6 g/L and this resulted in a pH range of 3.5–3.8 across the treatments (Table S2). The alcohol concentration of the finished wines was found to correlate with the must TSS pre-ferment and the results are included as Supplementary Information (Table S2). Outcomes of the treatments in terms of the wine alcohol concentration were close to what was reported previously using results from the end of fermentation [6], with the exception that alcohol concentration was found to be significantly different between the DA-1 and DA-2 treatments. In finished wines, significant differences in wine alcohol were found between the two levels of water addition, when the comparison was made for either the substitution or the direct addition treatments, respectively.

An increase in wine tannin, non-bleachable pigments and wine colour density was found with the transition of the harvest date from H-1 to H-3, and was also described in a previous publication based on the same experiment, reporting data for young wines [6] (Table 1). It was also previously reported that a reduction in wine tannin concentration, non-bleachable pigments and colour density were brought about by both water addition treatments [6] and this effect was also found to be maintained at 12 months. Small increases in polymeric pigments occurred with ageing for all wines relative to the previous report [6], together with losses in monomeric anthocyanin, which were expected effects. It was interesting to note that the saigneé treatment with the lowest juice run-off (RO-2) was equivalent to the H3 control for tannin and non-bleachable pigment measures, which were previously observed to be higher for RO-2 [6]. This was relevant, since other researchers have also reported a reduction or the loss of initial gains in wine phenolics by the use of saigneé, after an ageing period [20].

Other compositional analyses performed at the 12-month time point after fermentation were wine polysaccharides, residual amino acids and fermentation products. In terms of total wine polysaccharides (Table 2), it was found that differences between treatments were minor, with the exception that the saigneé treatment with the largest juice run-off (RO-1) had an elevated polysaccharide concentration relative to some treatments, but without clear treatment-specific effects. Differences in the polysaccharide concentration of RO-1 were evident for most of the component monosaccharides, indicating that the higher polysaccharide concentration was not driven by differences in specific polysaccharide sub-classes. Importantly, the difference between RO-1 and the H-3 wine was not significant for either total polysaccharides, or the component monosaccharides. Differences in the total polysaccharides were not found between the wines made from the fruit collected at the three harvest time points. However, from H1 to H3, increases in the component monosaccharides glucose, xylose and arabinose were found. Glucose-based polysaccharides could have been derived from either yeast or grape, while the differences in xylose- and arabinose-based polysaccharides indicate an increased extraction and retention of grape hemicellulose components. Previous studies on the Cabernet Sauvignon grape variety have reported the variable effects of grape ripening on grape-derived polysaccharides in wine with a change in the harvest date [3,21]. This potentially indicates that seasonal and site-specific effects exist in terms of polysaccharide extractability from the grape during ripening. Both studies showed that yeast-derived mannoproteins increased in wine with later harvests, which was not clearly shown in the current study on Shiraz. The study by Schelezki et al. [3] included a water addition component in addition to investigating ripening, and found that the polysaccharide concentration was increased only when the juice was substantially substituted with water (44% substitution), but no change was found for smaller quantities of water addition, similar to those applied in our study (<27% substitution).

Wine fermentation volatiles (Table 3) generally did not differ widely between the treatments, as was observed for polysaccharides, however a sub-set of variables were significantly different. The H-2 wines differed significantly from the other treatments, including H-1, being higher in the C-6 products hexanol and hexyl acetate, as well as in 2-phenylethyl acetate. The H-1 wines and the H-3 control, together with the water-addition treatments, were similar in their fermentation volatile composition, but were somewhat higher in the decanoic and octanoic volatile acids and their corresponding esters. Both of the saigneé treatments were lower in the above-mentioned compounds and somewhat higher in butanol. In another study on water addition in Shiraz must with a lower initial TSS [7], juice substitution with water was found to reduce acetate esters of higher alcohols in particular, as well as certain other esters, but these were not significantly affected in our study.

Generally, the concentration of residual amino acids is not considered to be relevant to wine sensory properties, being thought to be largely consumed by yeast as a nitrogen source during fermentation, with the exception of proline [22]. However, our recent work (unpublished) has indicated that some amino acids are found in wine at concentrations which approach or exceed reported taste thresholds, in particular glutamic acid which could potentially relate to “savoury” taste properties [23], although it is unlikely to be in salt form at wine pH. The amino acid profile was determined for all treatments and is included as Supplementary Information (Table S3). Differences in residual amino acids were found related to the harvest date, primarily driven by increases in proline, which is known to increase with ripening and is expected to remain in wine post-fermentation. Wines made from the earliest harvest were clearly separated from all of the other treatments, having higher residual isoleucine, phenylalanine, threonine, leucine, tyrosine and lysine levels but lower levels of other amino acids, including proline. For the remaining amino acids, water addition generally decreased all residual amino acids in wine, including proline. With the exception of proline, residual amino acids were lowest in wines made from the second harvest (H-2).

### 3.2. Outcomes of Grape Ripening, Water Addition and Saigneé on Wine Sensory Properties

From the ANOVA performed on the sensory data for the wines, 23 attributes rated by the panel differed significantly (*p* < 0.05) between the treatments, with a further five attributes being close to significant (*p* ≤ 0.10) (Table S1). There were significant winemaking replicates nested in treatment effects for the attributes “opacity”, “menthol aroma”, “savoury aroma”, “saltiness” and “savoury flavour”. These significant (*p* ≤ 0.05) effects indicated variation between some winemaking replicates within some treatments. The mean values of the sensory attributes for each treatment, together with the statistical results, are shown as Supplementary Information (Tables S1 and S4).

**Figure 1:**
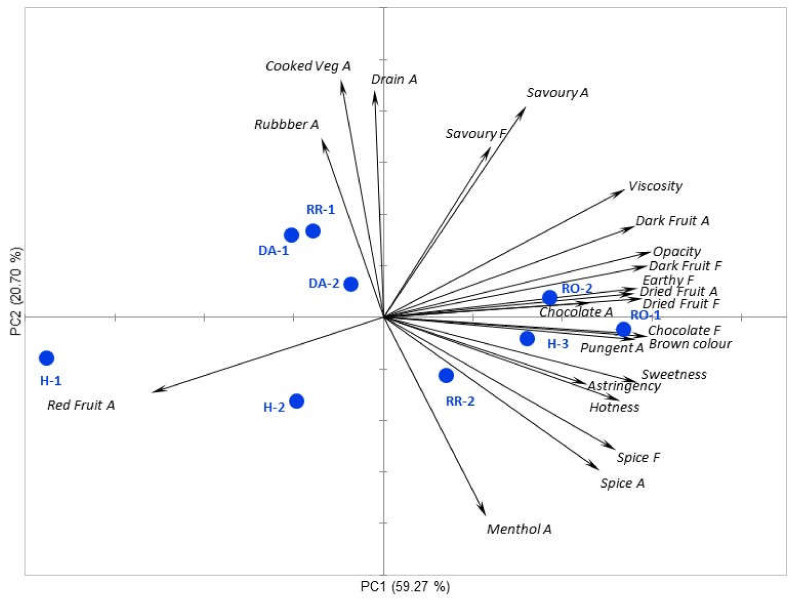
Principal component biplot of significant (*p* < 0.05) and close to significant sensory attributes (*p* < 0.10) for the harvest time and water addition winemaking treatments determined in Shiraz wines at 12 months post-fermentation. A: Aroma, F: Flavour, H-1, H-2, H-3 = harvest-1, harvest-2 and harvest-3; DA-1, DA-2 = direct water addition treatment 1 and direct water addition treatment 2; RR-1, RR-2 = juice substitution treatment-1, juice substitution treatment 2; RO-1, RO-2 = saigneé treatment 1 and saigneé treatment 2.

The PCA of the sensory data showed that the wines could chiefly be defined by a number of attributes associated with the harvest date, the majority of the variance explained by PC1 (59%, Figure 1). The H-3 treatment had high “dark fruit” and “dried fruit” aromas/flavours, “hotness”, “viscosity”, “astringency” and “opacity” (among other attributes), while the wines made from the earlier harvests were rated lower in these attributes and had progressively higher scores for “red fruit” aroma (Figure 1). The saigneé treatments were not substantially different in terms of their sensory properties, and were grouped with the H-3 control, but had a higher intensity of the attributes which were positively loaded on PC1, notably “dried fruit”, “chocolate” and “earthy” (Table S4), with the RO-1 treatment being highest in these attributes, and the RO-2 treatment was higher than the H-3 treatment, although generally the differences were not statistically significant. For the treatments where water was added to the 15.5° Baume must, the effect of water addition on sensory attributes was greater as the quantity of water added increased, in contrast to the effects observed on wine phenolics (Table 1). Water addition lowered the perceived intensities of the above-mentioned attributes associated with the H-3 control and the saigneé wines and increased the perception of “red fruit” aroma. The method of dilution was more important where the lower level of water addition was considered than in treatments which had the greater quantity of water. In this case, it was observed that the RR-2 treatment was more similar to the H-3 control than the DA-2 treatment. Both DA water addition treatments and the high juice substitution treatment RR-1 were separated from the other treatments on PC2, which was positively associated with “cooked vegetable”, “drain” and “savoury” attributes, and to a lesser extent negatively associated with a “menthol” aroma and “spice” attributes. The development of “cooked vegetable” and “drain” attributes in these wines could be considered to be undesirable sensory attributes. These attributes are potentially due to the presence of sulfur-containing volatiles with low molecular weights (reductive) which were not measured in this study. It is important to note that this issue was not previously found for Cabernet Sauvignon and Shiraz wines which received even greater levels of water addition [7,8,9] and further investigation is therefore needed before firm recommendations on water addition can be provided to wine producers. Generally the RR-2 treatment which received the smaller addition of water as a juice substitution without changing the solids:liquid ratio was more similar to the H-3 control and did not have an increase in potentially undesirable attributes associated with water addition.

### 3.3. Partial Least Squares Regression to Predict Wine Sensory Properties from the Wine Chemical Composition

In order to gain a better understanding of how the chemical composition of the wine influenced the wine sensory properties, a PLS regression analysis was performed (Table 4). Only wine compositional variables that were significantly different between treatments based on the ANOVA were used in the model. The residual amino acid data were not included in the final model, since it was not correlated with “savoury” attributes, and did not improve models for the prediction of other sensory attributes when combined with the remaining volatile and non-volatile data. From the PLS analysis, not all of the variables which were relevant in distinguishing the treatments by PCA (Figure 1) were well-modelled by the compositional data, for example the “hotness”, “astringency”, and “savoury” aromas and flavours, or the aromas of “drain” and “cooked vegetable”. It was unexpected that “hotness” in particular was not well described by the compositional data given that the differences in wine alcohol between the harvest date and water addition treatments were significant. A linear regression analysis of the “hotness” and alcohol concentration gave an R^2^ of 0.76 (data not shown), indicating a positive relationship when the data were assessed independently of other wine compositional attributes.

**Figure 2:**
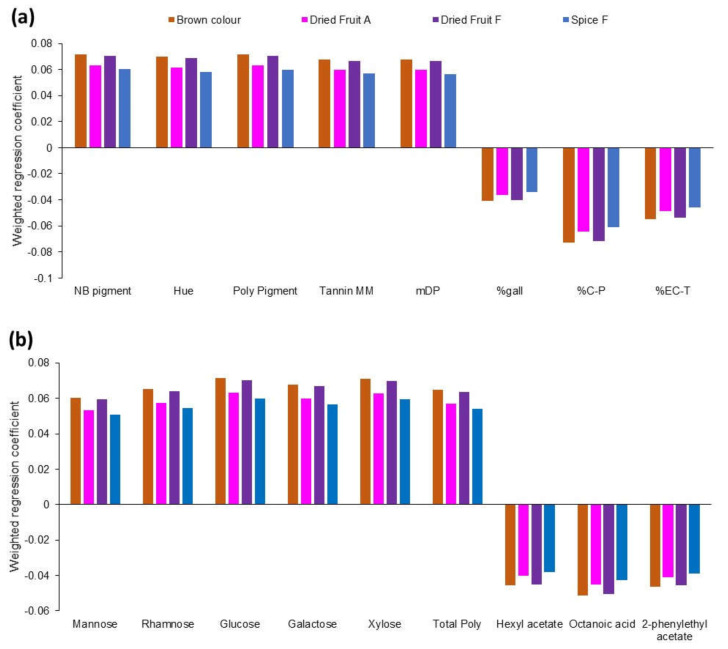
Weighted regression coefficients from partial least squares regression analysis of well-modelled sensory attributes (A, aroma; F, flavour) from a sub-set of significant variables, (**a**). Phenolic measures (NB pigment, pigments which are non-bleachable, or resistant to bleaching by bisulfite; poly pigment, polymeric pigment; MM, average molecular mass of tannin determined by subunit composition; mDP, mean degree of tannin polymerisation; %gall, degree of tannin galloylation; %C-P, proportion of catechin extension subunits in tannin; %EC-T, proportion of epicatechin terminal subunits in tannin) and (**b**). Polysaccharide-associated monosaccharides (poly, polysaccharide) and fermentation volatiles.

Certain attributes could be well-modelled in PLS by the wine compositional data, as evidenced by the high R^2^ values for model validation, together with low root mean square errors (RSME) of prediction. Some differences in the model parameters were observed if the model was developed using the whole wine chemistry dataset, or a sub-set of significant variables identified by an uncertainty test. In particular, the “brown colour”, “dried fruit” aroma and flavour, and “spice” flavour were particularly well-modelled using only a sub-set of significant variables. All of the above-mentioned sensory attributes were associated positively with multiple phenolics measures, including the wine hue (brownness), non-bleachable pigments, and tannin size (molecular mass and degree of polymerisation) (Figure 2). The proportion of tannin galloylation may be related to higher levels of extraction from the seed during fermentation, and this was higher in more brown wines, and higher in wines with “dried fruit” attributes and “spice” flavour. Wine polysaccharides, including both grape- and yeast-derived monosaccharides, were positively associated with these four sensory attributes. Three fermentation volatiles were relevant to the PLS model, octanoic acid, hexyl acetate and 2-phenylethyl acetate, and were all lower in wines with greater expressions of the above-mentioned sensory attributes. Interestingly, although the model was not as strong as for the four attributes discussed here, these three fermentation-derived compounds were all positively associated with the “red fruit” attribute in wines prepared from fruit harvested earlier and water-addition wines (weighted regression coefficient >0.03).

Hexyl acetate is thought to contribute a red fruit aroma to Cabernet Sauvignon wine [24,25] and to be associated with C6 precursors which are elevated in grapes of a lower ripeness. In another study on Shiraz, both hexyl acetate and 2-phenylethyl acetate were reduced by water addition to must (substitution) [7], and as mentioned previously these compounds were not affected by water addition in the current study. However, the previous study [7] was conducted on wines made from Shiraz fruit harvested at 14.5° Baume, equivalent to H-2 in the current study. It is possible that a peak in precursors for these acetate esters might occur earlier in the ripening of Shiraz, as was observed in the current results (Table 3). In this instance, water addition may have a greater effect on reducing the key fermentation-derived volatiles associated with the earlier harvest point.

Increased hexyl acetate was associated with the red fruit character of the water addition treatments in the PLS (although it was not necessarily statistically significant by ANOVA) in this research, which may indicate that changes in the conditions of fermentation associated with a lower must sugar (and final wine alcohol) have facilitated its formation. It is also well known that higher concentrations of 2-phenylethyl acetate can enhance the fruitiness of red wines [26], however here it was demonstrated for the first time that a negative correlation with “dark fruit” and “dried fruit” attributes exists for Shiraz, while positively associated with a “red fruit” character. Octanoic acid was well above the sensory threshold [27] but is thought to present a mild, somewhat rancid, yet fruity odour and its role as a potential impact odorant would require further investigation. In interpreting the current results, other relevant volatiles which are expected to increase with Shiraz shrivel, such as norisoprenoids and γ-nonalactone [10], were not measured. Therefore, in interpreting the relationships described in the PLS regression, other classes of compounds may also have contributed significantly to introduce changes to key sensory attributes.

To conclude, many of the other sensory variables were correlated, as shown in the PCA (Figure 1) and the same wine compositional variables were relevant in the prediction of these attributes as well. The regression coefficient data for the model developed using the sub-set of significant variables are shown as Table S5.

## 4. Conclusions

This project aimed to address the issue of grape ripeness as a first response to the current issues raised by vintage compression. The experimental aim was to discover whether the changes in wine sensory attributes achieved by extending the “hang time” would be retained following water addition, relative, in particular, to wines made from earlier-harvested fruit. The study highlighted that delaying the harvest of Shiraz introduced meaningful increases in wine phenolics, as also reported previously [6]. Water addition treatments to the later-harvest musts consistently resulted in wines with enhanced phenolic characteristics, colour and important sensory properties when compared with wines made from the earlier harvests.

While these results indicated that water addition is a simple strategy to control wine alcohol in later harvested Shiraz grapes, caution should be applied in practice. It might be expected that direct water addition would potentially increase the final wine volume. However, losses in berry weight (shrivel) often occur with an extended “hang time” [3,4,10], and this was also confirmed for the current study, as published previously [6]. Hence, water addition might recover volume losses caused by shrivel, but will not necessarily increase the wine volume relative to what may have been achieved by harvesting sooner. Therefore, the potential gains in wine sensory quality achieved by an extended “hang-time” need to therefore be carefully considered when using water addition as a remediation strategy to compensate for fruit volume loss and TSS increases.

A knowledge gap exists with respect to the grape volatile precursors which give rise to “dried fruit” characters in wines as the grapes begin to dehydrate, and whether these attributes are in fact acceptable to wine consumers. However, it was found that together with the observed increase in “dried fruit” attributes in wines made from riper fruit, other sensory attributes which might be considered beneficial, such as “chocolate” and “dark fruit”, also increased. Moreover, these attributes were retained in musts which received water addition in comparison with wines prepared from earlier-harvested fruit. This might present a relative gain in the wine sensory quality, but an important additional consideration was that “reductive” off-odours developed in response to higher water addition volumes. Further research to understand the development of off-odours in response to pre-ferment water addition is recommended, based on our findings. Therefore, the current recommendation is that the quantity of water addition to must is minimised, where possible, in a commercial context.

Before the use of water addition becomes more widespread as a means to alleviate issues with high TSS musts, research could also continue to focus on the consequences of water addition across a wider range of varieties which are relevant to Australian production, for example white grape varieties such as Chardonnay. Furthermore, for red winemaking, alternative maceration strategies such as ultrasound, pulsed electric field or “accentuated cut edges” (ACE) [28,29,30,31] could be explored, which might improve the phenolic concentration and sensory profile of wines made from less mature fruit. Hence, wine alcohol might be reduced by simply harvesting earlier. By harvesting earlier, losses in yield, increases in “over-ripe” characters with an extended “hang time”, and the requirement for the water addition or other alcohol reduction techniques could be avoided.

## Figures and Tables

**Table 1:** Wine phenolic concentration, tannin composition and colour properties in Shiraz wines prepared following harvest time, saigneé and water addition treatments 12 months after fermentation ^†^.

Phenolic Measure	Unit	H-1 ^‡^	DA-1	RR-1	RO-1	H-2	DA-2	RR-2	RO-2	H-3	ANOVA *p* Value
Total phenolics	(A.U.)	24.43 ± 0.52 ^f^	32.74 ± 0.63 ^d^	32.95 ± 0.46 ^d^	38.79 ± 1.13 ^a^	28.38 ± 1.05 ^e^	34.62 ± 0.89 ^c^	33.91 ± 0.58 ^cd^	36.84 ± 0.91 ^b^	36.75 ± 0.52 ^b^	<0.0001
Colour measures											
Colour density	(A.U.)	7.64 ± 0.14 ^e^	10.58 ± 0.29 ^c^	10.73 ± 0.17 ^c^	12.64 ± 0.49 ^a^	9.47 ± 0.21 ^d^	11.03 ± 0.29 ^c^	10.85 ± 0.19 ^c^	11.82 ± 0.40 ^b^	11.75 ± 0.23 ^b^	<0.0001
Hue	Ratio	0.54 ± 0.01 ^e^	0.57 ± 0.01 ^d^	0.56 ± 0.00 ^de^	0.66 ± 0.02 ^a^	0.54 ± 0.01 ^e^	0.59 ± 0.00 ^c^	0.59 ± 0.00 ^c^	0.64 ± 0.00 ^b^	0.64 ± 0.00 ^b^	<0.0001
Total Anthocyanin	(mg/L)	340 ± 10.66 ^d^	442 ± 16.85 ^b^	452 ± 5.42 ^ab^	438 ± 27.84 ^b^	411 ± 10.62 ^c^	472 ± 10.71 ^a^	453 ± 9.1 ^ab^	439 ± 14.04 ^b^	438 ± 8.94 ^b^	<0.0001
Non-bleachable pigment	(A.U.)	1.35 ± 0.12 ^e^	1.99 ± 0.09 ^c^	2.01 ± 0.08 ^c^	3.14 ± 0.26 ^a^	1.58 ± 0.05 ^d^	2.04 ± 0.10 ^c^	2.10 ± 0.03 ^c^	2.75 ± 0.07 ^b^	2.62 ± 0.05 ^b^	<0.0001
Polymeric pigment	(mg/L)	10.9 ± 0.63 ^g^	18.4 ± 0.56 ^e^	19.6 ± 0.29 ^e^	36.2 ± 1.96 ^a^	15.5 ± 0.18 ^f^	20.1 ± 1.15 ^de^	22.1 ± 0.35 ^cd^	31.9 ± 0.48 ^b^	24.5 ± 0.1 ^c^	<0.0001
Tannin concentration and composition											
Tannin	(mg/L)	232 ± 20.01 ^e^	539 ± 27.95 ^b^	513 ± 2.29B ^c^	82 ± 73.22 ^a^	369 ± 29.37 ^d^	545 ± 44.99 ^b^	548 ± 12.53 ^b^	742 ± 73.40 ^a^	723 ± 7.29 ^a^	<0.0001
Molecular mass (subunit) ^§^	(g/mol)	1144 ± 32 ^f^	1372 ± 32 ^d^	1351 ± 19 ^d^	2026 ± 65 ^a^	1183 ± 5 ^ef^	1333 ± 44 ^de^	1441 ± 14 ^cd^	1770 ± 15 ^b^	1541 ± 22 ^c^	<0.0001
Molecular mass (50% GPC) ^§^	(g/mol)	1444 ± 50 ^abc^	1457 ± 25 ^abc^	1430 ± 17 ^bc^	1537 ± 71 ^a^	1473 ± 25 ^abc^	1423 ± 35 ^c^	1453 ± 25 ^abc^	1477 ± 15 ^abc^	1531 ± 9 ^ab^	<0.01
mDP ^††^	no units	3.79 ± 0.12 ^f^	4.52 ± 0.11 ^d^	4.43 ± 0.07 ^d^	6.64 ± 0.20 ^a^	3.90 ± 0.03 ^ef^	4.38 ± 0.14 ^de^	4.72 ± 0.04 ^cd^	5.81 ± 0.06 ^b^	5.05 ± 0.07 ^c^	<0.0001
Mass conversion^‡‡^	(%)	13.68 ± 0.64 ^d^	26.01 ± 0.60 ^a^	27.11 ± 0.97 ^a^	20.48 ± 1.40 ^bc^	17.57 ± 0.67 ^cd^	24.35 ± 0.87 ^ab^	24.69 ± 0.10 ^a^	17.92 ± 0.77 ^c^	17.50 ± 0.57 ^cd^	<0.0001
Galloylation ^§§^	(%)	5.80 ± 0.90 ^ab^	5.65 ± 0.34 ^ab^	5.88 ± 0.08 ^ab^	4.88 ± 0.13 ^ab^	6.94 ± 0.67 ^a^	6.11 ± 0.20 ^ab^	5.49 ± 0.07 ^ab^	4.51 ± 0,25 ^b^	6.25 ± 0.34 ^ab^	<0.05
Epigallocatechin (ext.) ^§§^	(mol %)	18.34 ± 1.69 ^d^	30.74 ± 0.93 ^c^	34.22 ± 0.49 ^bc^	44.88 ± 2.0 ^a^	17.00 ± 1.23 ^d^	30.47 ± 2.46 ^c^	40.96 ± 1.29 ^ab^	46.10 ± 1.49 ^a^	33.08 ± 1.69 ^c^	<0.0001
Catechin (ext.) ^§§^	(mol %)	4.16 ± 0.23 ^a^	3.43 ± 0.09 ^abc^	3.48 ± 0.15 ^abc^	2.12 ± 0.12 ^e^	3.55 ± 0.18 ^abc^	3.59 ± 0.17 ^ab^	3.14 ± 0.01 ^bcd^	2.42 ± 0.23 ^de^	2.78 ± 0.12 ^cde^	<0.0001
Epicatechin (ext.) ^§§^	(mol %)	47.31 ± 1.03 ^a^	39.23 ± 0.77 ^b^	35.17 ± 0.90 ^bc^	34.06 ± 1.70 ^bc^	49.23 ± 1.30 ^a^	38.69 ± 1.76 ^b^	30.80 ± 1.05 ^c^	30.91 ± 1.94 ^c^	39.55 ± 1.44 ^b^	<0.0001
Epicatechin-gallate (ext.) ^§§^	(mol %)	3.75 ± 0.64	4.43 ± 0.29	4.57 ± 0.08	3.86 ± 0.02	4.57 ± 0.28	4.36 ± 0.15	3.92 ± 0.07	3.37 ± 0.25	4.78 ± 0.26	ns
Catechin (ter.) ^§§^	(mol %)	16.48 ± 0.24 ^a^	13.07 ± 0.25 ^b^	12.92 ± 0.33 ^b^	8.63 ± 0.49 ^c^	16.74 ± 0.29 ^a^	13.19 ± 0.39 ^b^	12.13 ± 0.17 ^b^	10.05 ± 0.20 ^c^	11.80 ± 0.06 ^b^	<0.0001
Epicatechin (ter.) ^§§^	(mol %)	7.90 ± 0.61 ^ab^	7.89 ± 0.28 ^ab^	8.33 ± 0.14 ^a^	5.43 ± 0.24 ^c^	6.55 ± 0.03 ^bc^	7.95 ± 0.39 ^ab^	7.47 ± 0.03 ^ab^	6.02 ± 0.04 ^c^	6.54 ± 0.21 ^ab^	<0.0001
Epicatechin-gallate (ter.) ^§§^	(mol %)	2.05 ± 0.32 ^ab^	1.21 ± 0.05 ^bc^	1.31 ± 0.04 ^bc^	1.02 ± 0.12 ^c^	2.37 ± 0.43 ^a^	1.75 ± 0.11 ^abc^	1.58 ± 0.01 ^abc^	1.14 ± 0.01 ^bc^	1.47 ± 0.11 ^abc^	<0.001

^†^ Data presented show mean values ± standard error; treatments were compared using one-way ANOVA where *p* < 0.05, *n* = 3, and differences between treatments were determined by a post-hoc Tukey’s test with significance indicated by different letters, ns = not significant; ^‡^ H-1, H-2, H-3 = harvest-1, harvest-2 and harvest-3; DA-1, DA-2 = direct addition treatment 1 and direct addition treatment 2; RR-1, RR-2 = juice substitution treatment-1, juice substitution treatment 2; RO-1, RO-2 = saigneé treatment 1 and saigneé treatment 2; ^§^ = molecular mass determined by using subunit composition from phloroglucinolysis (subunit) or determined at 50% elution by gel permeation chromatography (50% GPC); ^††^ = mean degree of polymerization; ^‡‡^ = mass conversion based on % recovery of proanthocyanidin by phloroglucinolysis as a proportion of tannin concentration by methyl cellulose precipitation; ^§§^ = molar proportion of subunits released by phloroglucinolysis: galloylation, total epicatechin-gallate including both extension and terminal subunits, ter. = terminal unit; ext. = extension unit.

**Table 2:** Wine total polysaccharide and composition of polysaccharide-derived monosaccharides (as mg/L) in Shiraz wines prepared following harvest time, saigneé and water addition treatments 12 months after fermentation ^†^.

Polysaccharide Component	H-1	DA-1	RR-1	RO-1	H-2	DA-2	RR-2	RO-2	H-3	ANOVA *p* Value
Total polysaccharide	655 ± 33 ^abc^	673 ± 2 ^abc^	674 ± 5 ^abc^	809 ± 17 ^a^	629 ± 25 ^c^	638 ± 69 ^bc^	763 ± 19 ^abc^	802 ± 1.5 ^ab^	771 ± 35 ^abc^	<0.01
Mannose ^‡^	141.18 ± 12 ^ab^	143.29 ± 1 ^ab^	130.9 ± 1 ^ab^	172.35 ± 5 ^a^	123.77 ± 6 ^b^	138.05 ± 16 ^ab^	155.43 ± 5 ^ab^	173.67 ± 3 ^a^	164.43 ± 5 ^ab^	<0.01
Rhamnose	46.86 ± 3 ^bc^	48.54 ± 1 ^bc^	51.72 ± 0.3 ^abc^	61.42 ± 1 ^a^	42.85 ± 2 ^c^	48.77 ± 3 ^bc^	55.63 ± 2 ^ab^	58.92 ± 1 ^a^	55.69 ± 2 ^ab^	<0.0001
Glucuronic acid	8.98 ± 1 ^d^	12.11 ± 1 ^bcd^	10.87 ± 1 ^bcd^	15.22 ± 1 ^a^	10.55 ± 1 ^cd^	12.15 ± 1 ^abcd^	13.53 ± 0.3 ^abc^	14.15 ± 1 ^ab^	14.87 ± 0.3 ^a^	<0.0001
Galacturonic acid	196.94 ± 3 ^a^	167.43 ± 8 ^ab^	191.8 ± 1 ^ab^	179.13 ± 4 ^ab^	175.73 ± 5 ^ab^	143.30 ± 21 ^b^	198.64 ± 1 ^a^	186.95 ± 2 ^ab^	182.72 ± 2 ^ab^	<0.05
Glucose	33.70 ± 2 ^c^	43.58 ± 1 ^bc^	41.73 ± 2 ^bc^	67.96 ± 1 ^a^	33.60 ± 2 ^c^	44.46 ± 5 ^bc^	53.71 ± 5 ^ab^	60.71 ± 3 ^a^	58.10 ± 2 ^ab^	<0.0001
Galactose	115.18 ± 9	120.59 ± 1	113.9 ± 1	145.39 ± 4	118.52 ± 6	117.15 ± 12	133.35 ± 5	142.19 ± 1	138.80 ± 6	<0.05
Xylose	8.41 ± 1 ^c^	10.95 ± 0.2 ^bc^	10.6 ± 0.1 ^bc^	17.27 ± 0.4 ^a^	8.10 ± 1 ^c^	11.52 ± 1 ^bc^	14.54 ± 1 ^ab^	16.65 ± 0.3 ^a^	16.01 ± 1 ^a^	<0.0001
Arabinose	103.50 ± 8 ^c^	127.02 ± 3 ^abc^	122.9 ± 2 ^abc^	150.52 ± 2 ^a^	115.75 ± 4 ^bc^	122.78 ± 11 ^abc^	137.79 ± 5 ^ab^	148.66 ± 2 ^a^	140.69 ± 7 ^ab^	<0.001

^†^ Data presented show mean values ± standard error, treatments were compared using one-way ANOVA; differences between treatments were determined by a post-hoc Tukey’s test with significance indicated by different superscript letters; H-1, H-2, H-3 = harvest-1, harvest-2 and harvest-3; DA-1, DA-2 = direct addition treatment 1 and direct addition treatment 2; RR-1, RR-2 = juice substitution treatment-1, juice substitution treatment 2; RO-1, RO-2 = saigneé treatment 1 and saigneé treatment 2; ^‡^ monosaccharide sugars released as polysaccharide components following acid hydrolysis.

**Table 3:** Fermentation-derived compounds in Shiraz wines prepared following harvest time, saigneé and water addition treatments 12 months after fermentation ^†^.

Fermentation-Derived Compound	Unit	H-1	DA-1	RR-1	RO-1	H-2	DA-2	RR-1	RO-1	H-3	ANOVA *p* Value
Acetate esters											
Ethyl acetate	mg/L	16.23 ± 0.33 ^c^	19.87 ± 1.9 ^abc^	19.2 ± 0.27 ^abc^	23.31 ± 1.90 ^a^	17.72 ± 0.24 ^bc^	21.58 ± 0.51 ^ab^	20.6 ± 0.68 ^abc^	23.28 ± 0.15 ^a^	23.01 ± 0.63 ^a^	<0.001
Hexyl acetate	µg/L	10.56 ± 0.62 ^bc^	10.19 ± 0.63 ^bc^	12.37 ± 0.57 ^b^	6.67 ± 1.11 ^d^	17.63 ± 0.41 ^a^	10.12 ± 0.44 ^bc^	10.98 ± 0.14 ^bc^	6.58 ± 0.26 ^d^	8.40 ± 1.16 ^cd^	<0.0001
3-Methylbutyl acetate	mg/L	0.61 ± 0.0 ^abc^	0.71 ± 0.02 ^ab^	0.69 ± 0.02 ^ab^	0.42 ± 0.09 ^c^	0.71 ± 0.02 ^ab^	0.75 ± 0.04 ^a^	0.79 ± 0.03 ^a^	0.51 ± 0.05 ^bc^	0.60 ± 0.06 ^abc^	<0.001
2-Methylbutyl acetate	µg/L	58 ± 4.42 ^bc^	82 ± 7.43 ^abc^	77 ± 2.90 ^abc^	54 ± 8.69 ^c^	76 ± 3.24 ^abc^	90 ± 7.65 ^a^	84 ± 4.68 ^ab^	61 ± 4.85 ^abc^	69 ± 6.80 ^abc^	<0.01
2-Phenylethyl acetate	µg/L	76 ± 2.68 ^b^	72 ± 4.19 ^bc^	71 ± 3.83 ^bc^	52 ± 4.32 ^c^	103 ± 4.06 ^a^	72 ± 4.12 ^bc^	77 ± 0.89 ^b^	61 ± 2.86 ^bc^	62 ± 7.66 ^bc^	<0.0001
Ethyl esters											
Ethyl butanoate	µg/L	103 ± 2.64	121 ± 4.41	127 ± 3.55	109 ± 15.9	122 ± 3.91	121 ± 11.85	137 ± 6.81	121 ± 7.87	117 ± 11.51	ns
Ethyl propanoate	µg/L	51.40 ± 2.52 ^ab^	50.77 ± 1.04 ^ab^	45.98 ± 0.64 ^b^	50.44 ± 1.90 ^ab^	54.71 ± 3.06 ^a^	46.99 ± 0.67 ^ab^	49.02 ± 0.42 ^ab^	53.15 ± 1.56 ^ab^	47.21 ± 2.01 ^ab^	<0.05
Ethyl hexanoate	µg/L	136 ± 0.04 ^cde^	150 ± 5.52 ^bcd^	159 ± 2.15 ^ab^	123 ± 6.37 ^e^	177 ± 5.42 ^a^	157 ± 0.08 ^abc^	159 ± 5.23 ^ab^	135 ± 1.01 ^de^	139 ± 6.81 ^bcde^	<0.0001
Ethyl octanoate	µg/L	109 ± 1.0 ^cd^	138 ± 2.23 ^a^	137 ± 0.6 ^ab^	105 ± 2.96 ^d^	136 ± 7.82 ^ab^	137 ± 1.77 ^ab^	128 ± 2.23 ^abc^	117 ± 2.35 ^bcd^	131 ± 8.12 ^ab^	<0.0001
Ethyl decanoate	µg/L	19.22 ± 1.88 ^b^	24.16 ± 0.45 ^ab^	23.41 ± 0.49 ^ab^	19.49 ± 0.58 ^ab^	20.87 ± 2.02 ^ab^	23.52 ± 0.82 ^ab^	20.49 ± 0.50 ^ab^	18.59 ± 0.15 ^b^	25.17 ± 1.79 ^a^	<0.01
Ethyl-3-methylbutanoate	µg/L	27.01 ± 2.17 ^b^	34.15 ± 1.0 ^ab^	34.28 ± 1.26 ^ab^	27.16 ± 0.51 ^b^	39.86 ± 4.01 ^a^	30.30 ± 0.84 ^b^	28.91 ± 0.66 ^b^	31.88 ± 0.64 ^ab^	28.37 ± 0.79 ^b^	<0.001
Ethyl-2-methylpropanoate	µg/L	62.73 ± 3.93	61.81 ± 3.62	61.61 ± 0.95	53.47 ± 4.77	60.53 ± 3.14	56.17 ± 2.90	55.52 ± 1.88	59.10 ± 6.53	59.76 ± 2.48	ns
Higher alcohols											
Butanol	mg/L	1.32 ± 0.06 ^c^	1.27 ± 0.07 ^c^	1.59 ± 0.07 ^bc^	1.98 ± 0.08 ^ab^	1.90 ± 0.05 ^ab^	1.20 ± 0.18 ^c^	1.84 ± 0.14 ^ab^	2.25 ± 0.04 ^a^	1.58 ± 0.14 ^bc^	<0.0001
2-Methylbutanol	mg/L	107.93 ± 1.70	120.47 ± 4.09	120.88 ± 4.78	106.02 ± 3.35	128.36 ± 4.23	117.84 ± 9.33	118.19 ± 3.36	118.05 ± 8.51	108.93 ± 2.52	ns
3-Methylbutanol	mg/L	233 ± 4.71	265 ± 15.04	253 ± 6.46	237 ± 9.87	267 ± 4.64	263 ± 19.06	257 ± 5.52	266 ± 17.75	239 ± 8.67	ns
Hexanol	mg/L	4.22 ± 0.29 ^b^	4.01 ± 0.24 ^b^	4.34 ± 0.20 ^b^	4.53 ± 0.33 ^b^	7.09 ± 0.49 ^a^	4.25 ± 0.26 ^b^	4.46 ± 0.10 ^b^	4.74 ± 0.29 ^b^	4.60 ± 0.23 ^b^	<0.0001
2-Phenylethanol	mg/L	50.59 ± 3.83 ^b^	58.35 ± 4.1 ^ab^	56.60 ± 2.81 ^ab^	52.62 ± 2.31 ^ab^	63.44 ± 1.54 ^ab^	54.67 ± 1.33 ^ab^	54.77 ± 1.56 ^ab^	64.90 ± 2.97 ^a^	57.75 ± 3.6 ^ab^	<0.05
2-Methylpropanol	mg/L	40.30 ± 0.78	38.63 ± 4.18	38.15 ± 3.10	37.62 ± 2.64	36.33 ± 3.59	40.48 ± 1.91	40.02 ± 1.95	41.67 ± 2.56	39.24 ± 2.12	ns
Volatile acids											
Butanoic acid	mg/L	1.02 ± 0.16	1.04 ± 0.01	1.08 ± 0.08	1.19 ± 0.13	1.10 ± 0.02	1.16 ± 0.05	1.08 ± 0.06	1.11 ± 0.07	0.97 ± 0.03	ns
Acetic acid	mg/L	273 ± 53.9	227 ± 38.4	272 ± 45.0	356.35 ± 35.9	214.60 ± 11.5	237.18 ± 13.0	281.54 ± 39.0	276.38 ± 3.19	283.34 ± 20.2	ns
Propanoic acid	mg/L	9.17 ± 0.75	11.38 ± 1.61	12.46 ± 1.14	12.56 ± 1.37	11.16 ± 0.70	12.69 ± 2.77	11.86 ± 0.91	13.02 ± 0.83	12.64 ± 1.42	ns
Hexanoic acid	mg/L	1.89 ± 0.22	1.96 ± 0.1	2.46 ± 0.42	1.68 ± 0.1	2.48 ± 0.12	1.80 ± 0.02	1.78 ± 0.04	1.78 ± 0.09	1.82 ± 0.22	ns
Octanoic acid	mg/L	1.07 ± 0.01 ^abc^	1.19 ± 0.01 ^ab^	1.28 ± 0.03 ^a^	0.84 ± 0.03 ^c^	1.14 ± 0.08 ^ab^	1.16 ± 0.06 ^ab^	1.06 ± 0.06 ^abc^	0.88 ± 0.02 ^c^	1.01 ± 0.09 ^bc^	<0.001
Decanoic acid	µg/L	154 ± 12.3 ^abc^	189 ± 6.89 ^ab^	200 ± 7.76 ^a^	151 ± 11.61 ^bc^	160 ± 9.27 ^abc^	188 ± 7.17 ^ab^	158 ± 7.24 ^abc^	138 ± 10.44 ^c^	176 ± 10.3 ^abc^	<0.05
2-Methylbutanoic acid	mg/L	1.04 ± 0.02	1.24 ± 0.06	1.16 ± 0.03	1.10 ± 0.06	1.05 ± 0.02	1.12 ± 0.03	1.17 ± 0.05	1.21 ± 0.08	1.03 ± 0.06	ns
3-Methylbutanoic acid	mg/L	1.20 ± 0.02	1.47 ± 0.11	1.39 ± 0.05	1.32 ± 0.08	1.37 ± 0.04	1.37 ± 0.09	1.47 ± 0.04	1.44 ± 0.03	1.31 ± 0.09	ns

^†^ Data presented show mean values ± standard error, treatments were compared using one-way ANOVA, ns = not significantly different, *n* = 3. Differences between treatments were determined by a post-hoc Tukey’s test with significance indicated by different superscript letters; H-1, H-2, H-3 = harvest-1, harvest-2 and harvest-3; DA-1, DA-2 = direct addition treatment 1 and direct addition treatment 2; RR-1, RR-2 = juice substitution treatment-1, juice substitution treatment 2; RO-1, RO-2 = saigneé treatment 1 and saigneé treatment 2.

**Table 4:** Partial least squares (PLS) regression analysis for the prediction of well-modelled wine sensory variables from wine chemical data, excluding amino acid analysis ^†^.

Sensory Attribute	Data Selected	PLS Model Parameters ^‡^				
		Factor No	R^2^_cal_	R^2^_val_	RMSE_cal_	RMSE_val_
Opacity	all variables	1	0.82	0.58	0.34	0.58
	sig var ^§^	3	0.96	0.58	0.15	0.58
Brown colour	all variables	1	0.92	0.86	0.10	0.14
	sig var	3	0.98	0.94	0.05	0.09
Red fruit aroma	all variables	1	0.61	0.49	0.16	0.21
	sig var	3	0.57	0.38	0.17	0.23
Dark fruit aroma	all variables	1	0.72	0.50	0.20	0.30
	sig var	3	0.96	0.58	0.07	0.28
Dried fruit aroma	all variables	1	0.83	0.73	0.13	0.18
	sig var	3	0.87	0.73	0.13	0.20
Spice aroma	all variables	1	-	-	-	-
	sig var	3	0.79	0.64	0.10	0.14
Earthy aroma	all variables	1	0.79	0.67	0.10	0.13
	sig var	3	0.78	0.62	0.09	0.14
Pepper aroma	all variables	1	0.70	0.53	0.07	0.09
	sig var	3	0.70	0.36	0.07	0.11
Pungent aroma	all variables	1	0.79	0.65	0.11	0.15
	sig var	3	0.87	0.62	0.08	0.16
Sweetness	all variables	1	0.81	0.71	0.10	0.14
	sig var	3	0.96	0.85	0.05	0.10
Viscosity	all variables	1	0.69	0.43	0.16	0.24
	sig var	3	0.89	0.35	0.09	0.25
Dark fruit flavour	all variables	1	0.73	0.43	0.23	0.38
	sig var	3	0.90	0.40	0.14	0.39
Dried fruit flavour	all variables	1	0.95	0.88	0.09	0.15
	sig var	3	0.95	0.90	0.09	0.14
Chocolate flavour	all variables	1	0.61	0.40	0.19	0.26
	sig var	3	0.79	0.38	0.14	0.26
Earthy flavour	all variables	1	0.74	0.53	0.14	0.21
	sig var	3	0.75	0.29	0.14	0.26
Spice flavour	all variables	1	0.63	0.51	0.16	0.21
	sig var	3	0.94	0.77	0.07	0.14

^†^ For the PLS model with all variables, Factor 1 explained 56% of the X variance and 57% of the Y variance; for the PLS model with a sub-set of significant variables, Factor 1 explained 84% of the X variance and 52% of the Y variance; Factor 2 explained a further 7% and 14% of the X and Y variance, respectively; ^‡^ PLS model parameters where cal = calibration, val = validation, RSME = root mean square error of prediction; ^§^ Sig var indicates a sub set of significant variables selected using an uncertainty test and high correlation loadings.

## Supplementary Materials

The following are available online at https://www.mdpi.com/2304-8158/9/9/1193/s1, Table S1. F-ratios, probability values, degrees of freedom and mean square error from the analysis of variance of the Shiraz 2017 wines; Table S2. Basic wine composition in Shiraz wines prepared following harvest time, saigneé and water addition treatments determined at bottling; Table S3. Residual amino acids in Shiraz wines prepared following harvest time, saigneé and water addition treatments; Table S4. Mean values for all sensory attributes which were identified as significant or close to significant descriptors of the harvest time, water addition and saigneé treatments in Shiraz; Table S5. Weighted regression coefficients from partial least squares regression analysis of all sensory attributes which were identified as descriptors of Shiraz wines prepared from different harvest dates, and which underwent water addition or saigneé treatments.
