# Supplementary material for: Pre-Fermentation Water Addition to High-Sugar Shiraz Must: Effects on Wine Composition and Sensory Properties
Source: Foods. 2020 Aug 28;9(9):1193. doi: 10.3390/foods9091193 (PMC7554985; doi:10.3390/foods9091193)
Supplement: Supplementary file 1 [file foods-09-01193-s001.pdf]

Table S1. F-ratios, probability values†, degrees of freedom (df) and mean square error (MSE) from the analysis of variance of the Shiraz 2017 wines.

Attribute	Treatment	WRep (Treatment)	Judge* Treatment	Judge*WRep (Treatment)	PRep (Treatment, WRep)	MSE
Opacity	46.35***	2.18**	1.91***	1.02	1.55*	0.519
Brown colour	10.81***	1.93*	3.41***	0.59	1.65*	0.369
Red fruit A	1.74ǂ	1.04	1.98***	0.84	1.79*	1.534
Confection A	0.53	1.06	1.59**	0.71	1.53*	1.822
Dark Fruit A	5.43***	0.80	2.17***	0.85	1.44	1.062
Dried Fruit A	2.82***	1.56	1.90***	0.78	1.19	1.633
Floral A	1.48	1.04	1.26	0.81	1.28	1.514
Spice A	3.48**	1.38	1.20	0.84	0.81	0.891
Chocolate A	2.66*	1.27	1.59**	0.84	0.87	1.857
Cooked veg A	1.76ǂ	0.73	1.25	1.01	1.56*	1.615
Earthy A	1.64	1.20	1.73**	0.81	0.69	1.320
'Sweet' plastic A	0.77	1.16	1.39*	0.90	0.99	1.327
Rubber A	1.84ǂ	1.04	1.07	0.79	0.71	0.642
Pepper A	1.31	0.90	1.16	0.86	1.02	0.837
Menthol A	2.42*	1.03	0.86	1.02	2.18*	1.286
Savoury A	3.18**	1.24	1.58**	0.85	1.88*	1.678
Pungent A	5.45***	0.80	0.92	0.99	0.97	0.780
Stalky A	0.78	0.49	1.22	1.06	1.43	1.560
Drain A	3.47**	1.12	1.34	0.90	1.79*	1.257
Sweetness	1.91ǂ	1.43	1.97***	1.18	0.83	0.883
Viscosity	7.49***	0.60	1.06	0.77	0.67	0.951
Acidity	1.15	0.50	1.08	0.82	0.91	0.735
Astringency	2.07*	1.28	1.31	0.94	1.34	0.828
Bitterness	1.42	0.85	1.05	1.00	0.87	1.465
Hotness	7.87***	0.84	1.04	0.91	1.29	1.173
Saltiness	0.91	1.73*	1.49*	0.88	1.75*	0.962
Savoury F	2.09*	1.89*	1.49*	0.51	1.30	1.158
Red Fruit F	1.31	0.92	3.44***	0.64	1.18	1.364
Confection F	1.12	0.79	1.48*	1.01	1.76*	1.488
Dark Fruit F	11.35***	1.30	1.73***	0.68	1.15	1.100
Dried Fruit F	3.85***	1.09	2.31***	0.79	0.73	1.640
Chocolate F	4.73***	0.64	1.20	0.79	0.68	1.487
Stalky F	1.04	1.25	1.98***	0.90	1.26	1.084
Earthy F	2.79**	0.88	1.74***	0.95	1.17	1.282
Spice F	2.63*	1.46	1.87***	0.91	1.16	1.140
Fruit AT	1.48	0.62	1.61**	0.83	1.27	0.739
df	8	18	80	180	27	270

A: aroma, F: Flavour, AT: After Taste. †Significance levels are as follows: * *P*≤0.05; ** *P*≤0.01; *** *P*≤0.001; ǂ *P*≤0.10. df = degrees of freedom. Judge effect was significant for all attributes (*P*<0.05), WRep = winemaking replicate, PRep = presentation replicate, J = Judge.

Table S2. Basic wine composition in Shiraz wines prepared following harvest time, saigneé and water addition treatments determined at bottling†.

Basic wine compositional measure	H-1	DA-1	RR-1	RO-1	H-2	DA-2	RR-2	RO-2	H-3	ANOVA P value:
Alcohol (%)	13.77 ± 0.03^{f}	14.70 ± 0.05^{d}	14.17 ± 0.03^{e}	16.27 ± 0.09^{a}	15.17 ± 0.03^{c}	15.37 ± 0.03^{bc}	15.43 ± 0.03^{b}	16.30 ± 0.06^{a}	16.37 ± 0.03^{a}	<0.0001
Residual sugar (g/L)	0.60 ± 0.00^{b}	0.73 ± 0.09^{ab}	0.63 ± 0.03^{ab}	0.27 ± 0.03^{c}	0.80 ± 0.06^{a}	0.10 ± 0.00^{c}	0.10 ± 0.00^{c}	0.27 ± 0.03^{c}	0.20 ± 0.00^{c}	<0.0001
pH	3.51 ± 0.01^{c}	3.56 ± 0.00^{c}	3.53 ± 0.01^{c}	3.77 ± 0.03^{a}	3.51 ± 0.01^{c}	3.65 ± 0.03^{b}	3.75 ± 0.02^{a}	3.71 ± 0.01^{ab}	3.76 ± 0.02^{a}	<0.0001
Volatile acidity (g/L)	0.33 ± 0.02^{bc}	0.33 ± 0.02^{bc}	0.31 ± 0.01^{c}	0.42 ± 0.03^{a}	0.33 ± 0.00^{bc}	0.32 ± 0.01^{bc}	0.32 ± 0.01^{bc}	0.34 ± 0.02^{bc}	0.39 ± 0.01^{ab}	<0.01
Free SO_2 (mg/L)	43.73 ± 0.27^{e}	48.0 ± 1.06^{abc}	46.67 ± 0.93^{cd}	48.53 ± 0.96^{abc}	45.60 ± 0.00^{de}	49.07 ± 1.21^{ab}	49.73 ± 0.13^{a}	47.20 ± 0.23^{bcd}	47.87 ± 0.53^{abc}	<0.001
Total SO_2 (mg/L)	100.33 ± 1.86^{a}	93.67 ± 2.96^{abc}	91.0 ± 2.08^{bcd}	86.67 ± 1.45^{cd}	97.33 ± 0.67^{ab}	93.67 ± 1.45^{abc}	91.33 ± 1.45^{bcd}	86.33 ± 0.33^{cd}	85.0 ± 0.58^{d}	<0.0001

†Data presented show mean values ± standard error, treatments were compared using one-way ANOVA, n=3. Differences between treatments were determined by a post-hoc Tukey's test with significance indicated by different superscript letters; H-1, H-2, H-3 = harvest-1, harvest-2 and harvest-3; DA-1, DA-2 = direct addition treatment 1 and direct addition treatment 2; RR-1, RR-2 = juice substitution treatment-1, juice substitution treatment 2; RO-1, RO-2 = saigneé treatment 1 and saigneé treatment 2;

Table S3. Residual amino acids in Shiraz wines prepared following harvest time, saigneé and water addition treatments[†]

Wine amino acids (mg/L)	H-1	DA-1	RR-1	RO-1	H-2	DA-2	RR-2	RO-2	H-3	ANOVA P value:
Asparagine	14.67 ± 0.52^{ab}	11.54 ± 0.34^{cd}	12.63 ± 0.75^{bcd}	16.81 ± 0.79^{a}	10.61 ± 0.34^{d}	14.01 ± 1.06^{abc}	12.72 ± 0.17^{bcd}	15.29 ± 0.12^{ab}	14.23 ± 0.43^{abc}	<0.0001
Glutamic acid	19.6 ± 2.28^{de}	21.45 ± 1.73^{cde}	20.03 ± 1.02^{de}	32.04 ± 1.08^{a}	16.26 ± 0.82^{e}	26.76 ± 1.06^{abc}	23.68 ± 1.78^{bcd}	28.66 ± 0.28^{ab}	31.41 ± 0.53^{a}	<0.0001
Serine	9.01 ± 1.29^{a}	7.24 ± 0.19^{ab}	6.64 ± 0.57^{ab}	9.03 ± 0.42^{a}	6.02 ± 0.65^{b}	8.96 ± 0.36^{a}	7.23 ± 0.32^{ab}	7.39 ± 0.26^{ab}	8.80 ± 0.07^{ab}	<0.01
Glycine	10.02 ± 0.91^{bc}	10.74 ± 0.54^{bc}	8.96 ± 0.33^{cd}	13.25 ± 0.31^{a}	7.36 ± 0.21^{d}	10.72 ± 0.85^{bc}	9.90 ± 0.30^{bc}	11.51 ± 0.16^{ab}	13.87 ± 0.29^{a}	<0.0001
Histidine	15.65 ± 1.73^{ab}	15.37 ± 0.55^{ab}	14.59 ± 0.26^{ab}	14.18 ± 0.07^{ab}	12.23 ± 0.53^{b}	15.84 ± 1.47^{ab}	13.51 ± 0.36 ^{ab}	12.55 ± 0.15^{ab}	16.31 ± 0.31^{a}	<0.05
Arginine	30.70 ± 5.78	20.74 ± 4.59	23.98 ± 3.31	29.86 ± 2.68	26.36 ± 1.71	29.32 ± 0.13	24.73 ± 0.66	24.82 ± 1.95	29.72 ± 3.46	ns
Threonine	55.60 ± 2.91^{b}	65.13 ± 3.66^{ab}	62.08 ± 3.53^{b}	73.60 ± 1.28^{a}	42.82 ± 1.47^{c}	61.69 ± 1.84^{b}	58.74 ± 1.46^{b}	65.56 ± 0.79^{ab}	62.62 ± 1.60^{ab}	<0.0001
Alanine	23.81 ± 1.68^{ab}	21.83 ± 1.14^{bc}	21.74 ± 0.98^{bc}	27.38 ± 0.69^{a}	17.43 ± 0.64^{c}	23.81 ± 1.13^{ab}	23.16 ± 0.31^{ab}	22.81 ± 0.39^{ab}	23.66 ± 0.76^{ab}	<0.001
Proline	843 ± 37^{f}	1403 ± 14^{cd}	1237 ± 57^{de}	1690 ± 63^{a}	1077 ± 10^{e}	1482 ± 17^{bc}	1403 ± 25^{cd}	1462 ± 57^{bc}	1646 ± 44^{ab}	<0.0001
Tyrosine	8.99 ± 0.87^{a}	6.23 ± 1.03^{ab}	5.23 ± 0.57^{b}	4.14 ± 0.29^{b}	4.67 ± 0.82^{b}	6.91 ± 0.91^{ab}	4.47 ± 1.12^{b}	4.25 ± 0.38^{b}	6.26 ± 0.19^{ab}	<0.001
Valine	6.84 ± 0.42^{a}	6.06 ± 0.34^{ab}	3.53 ± 0.56^{d}	4.09 ± 0.27^{bcd}	4.37 ± 0.37^{bcd}	5.63 ± 0.71^{abc}	3.42 ± 0.14^{d}	4.03 ± 0.32^{cd}	7.31 ± 0.10^{a}	<0.0001
Methionine	4.75 ± 0.41^{b}	8.19 ± 0.15^{a}	4.79 ± 0.47^{b}	5.26 ± 0.13^{b}	3.45 ± 0.13^{c}	5.75 ± 0.14^{b}	5.09 ± 0.13^{b}	4.90 ± 0.14^{b}	8.39 ± 0.20^{a}	<0.0001
Isoleucine	13.36 ± 2.65^{a}	6.71 ± 0.49^{b}	7.11 ± 0.56^{ab}	10.11 ± 0.10^{ab}	8.26 ± 0.62^{ab}	8.95 ± 2.75^{ab}	8.42 ± 0.56^{ab}	11.18 ± 0.31^{ab}	7.52 ± 0.13^{ab}	<0.05
Leucine	18.41 ± 1.89^{a}	10.12 ± 0.85^{bc}	9.63 ± 0.88^{bc}	13.17 ± 0.08^{abc}	8.03 ± 0.98^{c}	14.11 ± 1.89^{ab}	10.86 ± 0.94^{bc}	13.97 ± 0.56^{abc}	13.73 ± 0.08^{abc}	<0.0001
Phenylanaline	14.44 ± 1.87^{a}	8.52 ± 0.67^{bc}	7.79 ± 0.25^{bc}	11.47 ± 0.34^{ab}	6.76 ± 0.29^{c}	10.47 ± 1.61^{abc}	9.09 ± 0.43^{bc}	10.59 ± 0.25^{abc}	10.54 ± 0.12^{abc}	<0.001
Lysine	26.27 ± 4.44^{a}	16.59 ± 0.44^{ab}	16.18 ± 1.22^{ab}	19.07 ± 1.24^{ab}	14.55 ± 2.40^{ab}	19.53 ± 2.82^{ab}	14.51 ± 0.39^{b}	17.43 ± 0.13^{ab}	17.18 ± 0.71^{ab}	<0.05
Total amino acids	1115 ± 51^{f}	1639 ± 21^{cd}	1462 ± 66^{de}	1973 ± 70^{a}	1266 ± 9^{ef}	1744 ± 4^{abc}	1633 ± 31^{cd}	1717 ± 57^{bc}	1918 ± 52^{ab}	<0.0001

†Data presented show mean values ± standard error, treatments were compared using one-way ANOVA, n=3. Differences between treatments were determined by a post-hoc Tukey's test with significance indicated by different superscript letters, ns = not significant; H-1, H-2, H-3 = harvest-1, harvest-2 and harvest-3; DA-1, DA-2 = direct addition treatment 1 and direct addition treatment 2; RR-1, RR-2 = juice substitution treatment-1, juice substitution treatment 2; RO-1, RO-2 = saigneé treatment 1 and saigneé treatment 2.

Table S4. Mean values for all sensory attributes which were identified as significant (P<0.05) or close to significant (P<0.01) descriptors of the harvest time, water addition and saigneé treatments in Shiraz (see Table S1 for statistics)

Treatment	H-1	DA-1	RR-1	RO-1	H-2	DA-2	RR-1	RO-1	H-3	HSD*
Opacity	4.12	5.78	5.99	6.88	5.28	5.91	6.13	6.67	6.63	0.54
Brown Colour	0.38	0.53	0.39	1.35	0.39	0.59	0.69	1.10	0.93	0.47
Red Fruit A	4.89	4.53	4.30	4.10	4.53	4.64	4.65	4.16	4.30	ns
Dark Fruit A	4.54	5.41	5.56	5.90	5.10	5.22	5.33	5.62	5.72	0.76
Dried Fruit A	2.93	3.18	3.56	3.88	3.11	3.20	3.65	3.69	3.58	0.84
Spice A	0.86	0.93	0.93	1.34	1.22	0.97	1.45	1.26	1.29	0.51
Chocolate A	2.77	2.97	3.63	3.76	3.22	3.30	3.59	3.37	3.27	0.85
Cooked Veg A	2.22	2.62	2.34	2.16	1.88	2.47	1.95	2.25	2.24	ns
Rubber A	0.62	0.66	0.65	0.47	0.38	0.53	0.51	0.73	0.39	ns
Menthol A	1.23	1.22	1.01	1.36	1.47	1.26	1.76	1.37	1.38	0.57
Savoury A	0.94	1.81	1.77	1.81	1.02	1.71	1.37	1.47	1.50	0.81
Pungent A	4.70	4.96	4.87	5.41	5.00	5.16	5.09	5.34	5.36	0.46
Drain A	0.62	1.41	1.00	0.74	0.72	1.04	0.56	1.09	0.65	0.67
Sweetness	0.98	1.13	1.11	1.65	1.24	1.06	1.43	1.61	1.42	ns
Viscosity	3.87	4.53	4.64	4.78	4.24	4.59	4.49	4.85	4.61	0.48
Astringency	4.85	5.02	4.85	5.20	5.21	5.07	4.98	5.34	5.28	0.55
Hotness	4.30	4.61	4.37	5.23	4.89	5.06	4.98	5.06	5.32	0.57
Savoury F	1.08	1.38	1.60	1.27	1.09	1.37	1.46	1.36	1.42	0.51
Dark Fruit F	4.49	5.54	5.52	5.96	5.28	5.56	5.76	5.94	6.02	0.62
Dried Fruit F	2.84	3.44	3.14	4.07	3.01	3.16	3.55	3.87	3.76	0.94
Chocolate F	2.48	2.68	3.05	3.46	3.08	3.23	3.19	3.40	3.20	0.65
Earthy F	2.85	3.46	3.14	3.69	3.20	3.55	3.52	3.71	3.71	0.79
Spice F	1.03	1.03	0.91	1.75	1.32	1.20	1.46	1.51	1.50	0.76

*HSD, honest significant difference

Table S5. Weighted regression coefficients from partial least squares (PLS) regression analysis of all sensory attributes which were identified as descriptors of Shiraz wines prepared from different harvest dates, and which underwent water addition or saigneé treatments (NB, non-bleachable; poly, polymeric; MM, molecular mass; mDP, mean degree of polymerisation; %gall, galloylation; -P, extension subunit; -T, terminal subunit; C, catechin; EC, epicatechin; sugars are polysaccharide-associated monosaccharides; poly, polysaccharide).

	Significant wine compostional measure used for PLS regression model															
Sensory attribute	Hexyl acetate	Octanoic acid	2-Phenylethyl acetate	NB pigment	Hue	Poly Pigment	Tannin MM	mDP	%gall	%C-P	%EC-T	Mannose	Rhamnose	Glucose	Galactose	Xylose	Total Poly
Opacity	-0.039	-0.043	-0.039	0.061	0.059	0.060	0.057	0.057	-0.035	-0.061	-0.046	0.051	0.055	0.060	0.057	0.060	0.055
Brown colour	-0.046	-0.051	-0.047	0.072	0.070	0.071	0.068	0.068	-0.041	-0.073	-0.055	0.060	0.065	0.072	0.068	0.071	0.065
Red Fruit A	0.033	0.037	0.034	-0.052	-0.051	-0.052	-0.050	-0.049	0.030	0.053	0.040	-0.044	-0.048	-0.052	-0.050	-0.052	-0.047
Dark Fruit A	-0.036	-0.041	-0.037	0.057	0.055	0.057	0.054	0.053	-0.032	-0.058	-0.043	0.048	0.052	0.057	0.054	0.056	0.051
Dried Fruit A	-0.040	-0.045	-0.041	0.063	0.061	0.063	0.060	0.060	-0.036	-0.064	-0.048	0.053	0.057	0.063	0.060	0.063	0.057
Spice A	-0.030	-0.034	-0.031	0.047	0.046	0.047	0.044	0.044	-0.027	-0.048	-0.036	0.040	0.043	0.047	0.044	0.047	0.042
Chocolate A	-0.027	-0.030	-0.027	0.042	0.041	0.042	0.039	0.039	-0.024	-0.042	-0.032	0.035	0.038	0.042	0.039	0.041	0.038
Cooked Veg A	0.002	0.003	0.002	-0.004	-0.004	-0.004	-0.003	-0.003	0.002	0.004	0.003	-0.003	-0.003	-0.004	-0.003	-0.004	-0.003
Pepper A	-0.038	-0.042	-0.039	0.060	0.058	0.059	0.056	0.056	-0.034	-0.060	-0.046	0.050	0.054	0.059	0.056	0.059	0.054
Menthol A	-0.012	-0.014	-0.012	0.019	0.019	0.019	0.018	0.018	-0.011	-0.019	-0.015	0.016	0.017	0.019	0.018	0.019	0.017
Savoury A	-0.021	-0.023	-0.021	0.032	0.031	0.032	0.030	0.030	-0.018	-0.033	-0.025	0.027	0.029	0.032	0.030	0.032	0.029
Pungent A	-0.040	-0.045	-0.041	0.063	0.061	0.063	0.060	0.060	-0.036	-0.064	-0.048	0.053	0.057	0.063	0.060	0.063	0.057
Drain A	0.002	0.003	0.003	-0.004	-0.004	-0.004	-0.004	-0.004	0.002	0.004	0.003	-0.003	-0.004	-0.004	-0.004	-0.004	-0.004
Sweetness	-0.042	-0.047	-0.043	0.066	0.064	0.066	0.063	0.062	-0.038	-0.067	-0.051	0.056	0.060	0.066	0.063	0.066	0.060
Viscosity	-0.035	-0.039	-0.035	0.054	0.053	0.054	0.051	0.051	-0.031	-0.055	-0.041	0.046	0.049	0.054	0.051	0.054	0.049
Astringency	-0.029	-0.033	-0.030	0.046	0.045	0.046	0.043	0.043	-0.026	-0.047	-0.035	0.039	0.042	0.046	0.043	0.045	0.042
Hotness	-0.033	-0.036	-0.033	0.051	0.050	0.051	0.048	0.048	-0.029	-0.052	-0.039	0.043	0.046	0.051	0.048	0.050	0.046
Savoury F	-0.011	-0.013	-0.011	0.018	0.017	0.018	0.017	0.017	-0.010	-0.018	-0.013	0.015	0.016	0.018	0.017	0.017	0.016
Dark Fruit F	-0.036	-0.040	-0.036	0.056	0.055	0.056	0.053	0.053	-0.032	-0.057	-0.043	0.047	0.051	0.056	0.053	0.056	0.051
Dried Fruit F	-0.045	-0.050	-0.046	0.071	0.069	0.070	0.067	0.066	-0.040	-0.072	-0.054	0.059	0.064	0.070	0.067	0.070	0.064
Chocolate F	-0.034	-0.038	-0.034	0.053	0.051	0.053	0.050	0.050	-0.030	-0.054	-0.041	0.045	0.048	0.053	0.050	0.052	0.048
Earthy F	-0.037	-0.042	-0.038	0.059	0.057	0.059	0.056	0.055	-0.034	-0.060	-0.045	0.050	0.053	0.059	0.056	0.058	0.053
Spice F	-0.038	-0.043	-0.039	0.060	0.058	0.060	0.057	0.056	-0.034	-0.061	-0.046	0.051	0.054	0.060	0.057	0.059	0.054
